# Mr. Cuming, on Mercury in Venereal Affections

**Published:** 1805-05-01

**Authors:** Ralph Cuming

**Affiliations:** Romsey


### *To the Editors of the Medical and Physical Journal.*

GENTLEMEN,

READING the paper with the signature of a Constant Reader, and entitled Diagnosis of Syphilis, in your Journal for December, 1804, led me into a train of thoughts, respecting the protracted and improper exhibition of mercury in venereal cases.

I do not mean this as any imputation against the ingenious author of the said paper, but quite the reverse; for it appears, from his candid statement of the case, that prudence, and the received opinion of many respectable authors, would induce any one to act in a similar manner, when unaided by that confidence which can only arise from extensive practice and observation; even with these advantages, the most skilful are sometimes deceived. It is not my intention to comment upon the case, as I am persuaded there is not much deference due to anonymous communications: I shall therefore confine myself chiefly to such remarks as have been the consequence of practical notes, included within the sphere of my own practice.

I have to observe, in the first place, that the diagnostic marks of syphilitic sores are not so well defined by the most accurate writers, neither indeed can be, as to enable a medical person to affirm positively, on inspection, that the criteria correspond so exactly with the general description of such sores, that they do infallibly originate from this source; far otherwise: I believe, the generality of practitioners will not be ashamed to acknowledge, with me, that they have been deceived plus qu'une fois.

I, the other day, was sent for to visit a girl, aged twelve years. On examining the fauces, I found the velum pendulum dulum palati, on each side ulcerated, exactly corresponding with the appearance which venereal sores assume: yet the remedies employed in cases of common cynanche soon effected a cure.

A young countryman placed himself under my care lately, for the cure of an ulcerated bubo. He informed me, that the medical gentleman who treated his complaint, prior to his application to me, had given him large quantities of pills, which, I suppose, were of calomel, as they had occasioned an incessant purging for some weeks. The patient, perceiving himself decline in health, and his sore growing larger, imagined his treatment was not agreeable to the rules of art, and changed his surgeon, which proved to be a fortunate circumstance, for a sinus had already formed itself, extending from the inferior border of his callous ulcer, along the edge of the scrotum, stretching towards the anus, in the direction of the rapha perinæi. I supposed, from the emaciated appearance of my patient, and excessive discharge from the groin, that his habit had either been supersaturated with mercury, or, that he was so much reduced, as to forbid the adoption of any treatment, save that which was calculated to restore lost stamina. On these premises, I desired him to leave off the pills, and, instead of a sparing diet, which he had been ordered to live upon, I caused him to pursue a plan diametrically opposite, and administered opium, in sufficient quantities to allay irritation, and suppress the purging: by the assistance of strong jellies, good port, ale, &c. he was easily restored to a better state of health. At the expiration of a month, finding the discharge from the bubo still ichorous, without any amendment in appearance, (it still exhibited an inflamed margin, with a white sloughy bottom) I thought myself justified in directing a drachm of ungt. hydrarg. to be rubbed into the thigh of the affected side; but he did not persevere in the use of this medicine for more than a week, when colloqnative night-sweats commenced, which determined me to abandon its use altogether, and confide in the tonic plan which I had set out on; aiding my efforts to heal this obstinate sore by the use of adhesive strips of plaster, *compresses,* and a tight roller, that was passed round the body and upper part of the thigh, in such a manner as to form a figure of eight, beginning by pinning it round the ilia, as is done in cases of amputation. I soon had the happiness to find this method succeed; the callosity disappeared, and the cicatrizing process was soon completed, by managing the bandage so as to to cause it to press immediately on the edges of the sore. And here I beg leave to observe, that there is frequently more done by the ingenuity of the surgeon, than by the most elaborate directions which can be given for the treatment of such cases; for I am fully persuaded, that two patients in an hospital, circumstanced exactly alike, if under the care of two dressers, who are not equally attentive to a number of nice and delicate alterations, necessary to be made in dressing, according to the various appearances which wounds put on, will experience great difference with respect to the termination of their complaints.

I wish to embrace this opportunity of recommending the application of graduated compresses, and firm bandaging in similar cases; conscious that great benefit is to be derived from this mode of dressing.

I believe several of the profession are in the habit of using mercury too liberally, in cases of bubo, very much to the prejudice of their patients, and disgrace of this remedy, which is still considered the sine qua non in venereal affections. An intimate medical acquaintance of mine, who had a chancre, succeeded by its usual concomitant, bubo, treated himself, secundum artem, by rubbing in a small quantity of ointment; but, being of an irritable habit, he was disappointed in his hopes of discussing it; the discharge was great, and it assumed a virulent aspect; he again had recourse to mercury, and took a combination of calomel with opium. Before he had used twenty grains, he found himself worse in every respect, and had profuse night-sweats, with a train of nervous disorders. Feeling himself staggered with respect to the nature of his complaints, and wavering concerning their proper treatment, he applied to a medical friend, who advised him, by all means, to desist from the further use of mercury, and to confide in a tonic course of medicine and diet, which soon restored him. From these and similar occurrences, which have come within the circle of my notice, I am inclined to suppose, that the sufferings of patients are frequently unnecessarily prolonged. Indeed, it would be fortunate if the evil ended here; but it is to be lamented, that deprivation of the power of procreation, premature death, and old age, may be included in the long catalogue of miseries, arising from the intemperate administration of this potent medicine. Yet, I must acknowledge, it is much easier to detect mal-practice, in this complaint, than to point out the precise point where to desist. The observations I have made, from a variety of of cases, embolden me to remark, agreeably to my own opinion, that local venereal complaints, such as chancres and buboes, require much less mercury than what is, in general, so liberally bestowed upon them. When we are enabled to discuss a bubo, I suppose, for the most part, the mercury arrests the enemy in his citadel, and, by destroying him there, prevents him from doing any further mischief; but some imagine, on his forsaking his position, that he has only retreated to appear in a more formidable manner; and, on the strength of this supposition, pursue a foe which they had already vanquished and totally annihilated. With respect to confirmed cases of lues venerea, the most expert are often left to grope in the dark, as if this path of science had been intentionally obscured, in order that the devotees of Venus should be severely punished for their illicit amours.

It is well known, that patients, ill of a confirmed lues, find their sufferings alleviated, when the mercury begins to act on the system; and when continued a certain time, so as to keep up a slight soreness of the mouth, by way of convincing us that the remedy pervades every part of the body, many of the complaints vanish, whilst others remain in statu quo, and not unfrequently become worse. When this is the case, it appears to be time to desist from its further use; more particularly, if languor, debility, and night-sweats commence. Should this opinion be founded on truth, it would appear, that more attention ought to be paid to the feelings of the patient, than the unfavourable aspect of certain remaining local complaints, that are continued from diseased action, brought on by accumulated irritability, the natural consequence of the powerful excitement which is produced by the mercury. Some people imagine, when there is a chance of erring, that it is adviseable to adhere to what they consider the safe side; thinking it best to push the mercury, fearing that the smallest relaxation may be productive of the most fatal consequences, when, in fact, they have nothing to dread: for if the system has been well saturated with the antidote, why not lie by on their oars a little, and see which way the tide sets. If the patient should become importunate, let the example of Hunter be imitated, by way of *a placebo*, give him a bread pill! I must now conclude the subject; which, I hope, will be resumed by some of your correspondents, whose time and talents will enable them to do more justice to it than,

Your's, &c.

*Romsey, March* 11, 1805. RALPH CUMING.

